# Relationship Between HBV Genotypes B, C and Follicular Helper T Cells in Patients with Chronic Hepatitis B and its Significance

**DOI:** 10.5812/hepatmon.6221

**Published:** 2013-01-20

**Authors:** Gu Xibing, Yang Xiaojuan, Wang Juanhua, Hua Zhong

**Affiliations:** 1 Department of Hepatology, Wuxi Hospital for Infectious Diseases, Wuxi, China

**Keywords:** Hepatitis B Virus, Genotype, T-Lymphocytes, Helper-Inducer, Receptors, Interleukin-21, T-Lymphocytes, Cytotoxic

## Abstract

**Background:**

Clinical observations have shown that patients infected with chronic hepatitis B virus (HBV) genotype C versus genotype B had a higher load of the virus, more serious illness, and poorer responses to antiviral therapy and prognosis. However, the disparity between the two has not been clarified.

**Objectives:**

To explore possible relationship between HBV genotypes B and C and peripheral blood follicular helper T cells (Tfh) and its significance in treating chronic hepatitis B (CHB) patients.

**Patients and Methods:**

One hundred and fifty CHB patients were enrolled into this study, including 70 cases infected with HBV genotype C and 79 cases with genotype B. One patient had suffered from both genotypes B and C. The levels of Tfh, also known as interleukin-21 (IL-21), HBV specific cytotoxic T lymphocytes (CTL), HBV DNA and alanine transaminase (ALT) were evaluated and compared in patients infected with genotype B and C.

**Results:**

Levels of Tfh, IL-21 and HBV specific CTL of patients infected with HBV genotype C were significantly lower than those of patients infected with HBV genotype B, P < 0.01. Levels of HBV DNA and ALT of patients infected with genotype C were significantly higher than those of the patients infected with HBV genotype B, P < 0.01.

**Conclusions:**

Compared with chronic hepatitis B (CHB) patients infected with genotype B, higher levels of serum HBV DNA, ALT and TBil of patients infected with HBV genotype C may be related to their lower level of peripheral blood Tfh, which may result in lower IL-21, and it may result in lower HBV specific CTL.

## 1. Background

Different genotypes of Hepatitis B virus (HBV) have shown different characteristics (1). HBV has 8 genotypes i.e., genotypes A to H. In China the dominant genotypes of HBV are genotypes B and C. The patients infected with HBV genotype C showed longer immune clearance period, higher serum HBV DNA level and activity of hepatic histology, had repeatedly or continuously fluctuating alanine transarninase (ALT), and their rate of response to interferon, lamivudine, and analogs of nucleotide was lower than those infected with HBV genotype B. After responding to antiviral therapy, patients infected with HBV genotype C had a lower persistent response rate than that of patients infected with HBV genotype B. It is not clear why such conditions exist in patients infected with different HBV genotypes. At present, the pathogenesis of hepatitis B is closely related to the pathological change of cellular immunity resulting from the process of clearing HBV from the body. The cellular immune system of clearing HBV is generally classified into specific and nonspecific (2), and specific cellular immune response of the body to the viruses is believed be an important factor for the body to eliminate viruses (3). Virus specific cytotoxic T lymphocytes (CTL) clear HBV mainly through two routes: cytotoxic or cytolytic and non-cytolytic (4), but in non-cytolytic route, the virus-specific T lymphocytes only remove viruses in target cells without causing injury to the target cell. Experimental studies showed that the non-cytolytic route might play a more important role in removing viruses by specific T cells (5). The effect of nonspecific cellular immunity on removing HBV is weaker and it may result in damage to the liver cells (6). Our former studies (7) demonstrated that compared with patients infected with HBV genotype B, the level of HBV specific CTL of patients infected with genotype C was significantly lower, resulting in a higher level of HBV DNA than in patients infected with HBV genotype B. Therefore, hepatic dysfunction in patients infected with HBV genotype C was more serious than that in those infected with HBV genotype B. When the activity of HBV specific CTL was inadequate, it could not remove HBV effectively, nonspecific CTLs generate inflammatory reaction of the liver, resulting in liver cell injury (8), while HBV specific CTL activity of patients infected with genotype C is lower, nonspecific CTL is activated, which aggravates injury to liver cells. It is not clear why the activity of HBV specific CTL in patients infected with HBV genotype C is lower than that of patients infected with genotype B. Recent studies revealed that there is a CD4 and CXCR5 double positive T cell subgroup in human peripheral blood, called follicular helper T cell (Tfh), which plays a major role in delivery of signals affecting cell division, helps to activate the B cell and adjust humoral immune response. It is clear that Tfh is a T cell subgroup responsible for helping B cell (9).

## 2. Objectives

Studies show that Tfh is associated with the occurrence of various diseases, including autoimmune disease, immune defects, infection and tumors. But there are only a few reports on changes and effects of Tfh in hepatitis B virus infection. Since Tfh can produce and secrete interleukin-21 (IL-21) (10, 11), which in chronic infection can increase proliferation of virus specific CTL to maintain long term effective antiviral immunity (12, 13). Therefore it is inferred that Tfh has certain relation with hepatitis B viral infection. We compared Tfh, IL-21, HBV specific CTL, nonspecific CTL, HBV DNA and degree of liver damage in patients infected with HBV genotypes B and C, in order to explore the possible association between HBV genotypes B and C and peripheral blood Tfh and its significance in CHB patients.

## 3. Patients and Methods

### 3.1. Patients

In total, 150 patients’ chronic hepatitis B (CHB) were enrolled into this study between January 2011 and June 2011 in Wuxi, China. Diagnosis of CHB was made according to the diagnostic criteria defined in 2005 Guidelines for Prevention and Treatment of Chronic Hepatitis B Stipulated by the Society of Hepatology and Society of Infectious Diseases, Chinese Medical Association (14). The inclusion criteria included adult patients who were positive for human leucocyte antigen (HLA)-A2 and HBV DNA (HBV DNA > 103 copies/ml), with various degrees of abnormal liver functions, age 18-60 years with no gender differences. The exclusion criteria included patients with hepatitis A, C, D or E, history of autoimmune diseases, alcoholism, and use of hepatotoxic agents, analog of nucleotide, interferon, other antiviral drugs or immune modulators. Of the 150 CHB patients, 107 (71.33%) were male, 43 (28.67%) were female, mean age was 35.17 ± 8.24 years. Thirty healthy blood donors were enrolled as healthy controls, of whom 22 (73.33%) were male and eight (26.67%) were female; the mean age was 35.87 ± 5.54 years. The present study was approved by the ethic committee of our hospital (No.20101215-3). 

### 3.2. Monoclonal Antibodies

The following labeled antibodies were used: 

a) PerCP/Cy5.5 conjugated mouse anti-human CD185 (clone: TG2/CCR5, isotype: mouse IgG2b, κ) (American Biolegend Company, San Diego, CA92121) and an isotype-matched negative control PerCP/Cy5.5 conjugated mouse antibody (IgG2b, κ, clone; G155-178) (Biolegend Company). 

b) Fluorescein isothiocyanate (FITC)-conjugated mouse anti-human CD4 (clone: RPA-T4, isotype: mouse IgG1, κ) (American Ebioscience Company,) and an isotype-matched negative control FITC -conjugated mouse antibody (IgG1, κ, clone; OPC-21) (Ebioscience Company).

c) FITC-conjugated mouse anti-human CD19 (clone: HIB19, isotype: mouse IgG1, κ) (American Ebioscience Company) and an isotype-matched negative control FITC-conjugated mouse antibody (IgG1, κ, clone; OPC-21) (Ebioscience Company).

### 3.3. Determination of Peripheral Blood Tfh

Whole blood 100 μl anticoagulated with heparin was added with 10 μl of CXCR5-PerCP/Cy5.5 monoclonal antibody and incubated at 4℃ for 30 min, then monoclonal antibodies to CD4-FITC and CD19-FITC 10 μl each were added; in the control tubes, control solutions with different colors (IgG1-FITC, IgG2b- PerCP/Cy5.5) corresponding to the monoclonal antibodies, were added and then were incubated at 4℃ for 30 minutes. After hemolysis, the contents of the tubes were washed with FACS cleaning solution and were fixed with FACS stationary liquid. The reagents for these tests were supplied by American Ebioscience Company and Biolegend Company. The Tfh cell was determined by Beckman-Coulter 3XL flowcytometer USA (15).

### 3.4. Determination of Peripheral Blood IL-21

A double antibody sandwich ELISA was applied; the instrument was Delang Enzyme Analyzer (Shanghai China), the reagent kit was supplied by American ADL Company. The testing was performed strictly according to instructions from the manufacturers.

### 3.5. Identification of HLA-A2 Allelotypes

Fresh blood specimen 100 μl anti-coagulated with heparin sodium was loaded into testing and control tubes. After adding 10 μl HLA-A2-PE and the same type control, the tubes were incubated at room temperature for 30 min in the dark and tested with Beckman-Coulter 3XL flowcytometer USA after hemolysis. Reagents were ordered from Proimmune, UK.

### 3.6. HBV-Specific CTL Test

HBV-specific CD8+ T cells measured by HLA-peptide tetramer flow cytometry, the principle of the method was that the tetramer is labeled with phycoerythin (PE), and CD8+ T cells from peripheral blood that are specific for an epitope identity and bind short peptide in the antigen channel through the T cell receptor (TCR); an FITC-labeled anti-CD8 is used to detect the CD8 + T cells, and a dual-color flow cytometry is used to detect HBV antigen-specific CD8+ T cells. The flow cytometry was performed as follows: test tubes were loaded with 10 μl PE labeled HLA-peptide tetramer, anti-CD8-FITC, CD3-PC5 and 1 μl HBV core 18-27 antigen peptide, added with 100 μl heparin anti-coagulated blood was added, mixed well and t incubated at room temperature for 20 minutes in the dark and tested with flowcytometer after hemolysis and rinsing. At the same time, parallel control test without adding the specific antibody to antigen peptide was also performed. CD3 + lymphocyte gate was used to count 5000 CD8 + cells, CD8 + and HLA-peptide tetramer double positive cells were counted as HBV-specific CD8 + cells, expressed as the percentage of total CD8 + cells counted. All reagents were purchased from Beckman Coulter (16).

### 3.7. Non-Specific CTL Test

Testing and control tubes were loaded with fresh EDTA-K2 anti-coagulated blood 100 μl, and added with 10 μl monoclonal antibodies (anti-CD8+-FITC and anti-CD28 + -PE produced by Beckman Coulter) and parallel nonspecific controls, respectively. The tubes were incubated at room temperature for 15 minutes in the dark and tested with flow cytometer after hemolysis (16).

### 3.8. HBV Genotype

PCR microplate nucleic acid hybrid-ELISA technology, gene expansion and proliferation instrument PE9600, PE U.S., reagents from Diagnosis Center of Basic Biomedicine of No. 1 Military Medical University (China) were used. Primer and probe: different sequences at front C zone were selected as PCR primer and probe of nucleic acid hybridization, six universal genotype sequences were used as primers of PCR and universal wrap probes, while different sequences at one part as chromogenic probes of various genotypes. Chromogenic probe 5’ labeled biotin was synthesized by Shanghai Bio-engineering Company; primer sequences： 1:5’-CCCTTCTTCGTCTGCG-GTTCC-3’ (nt1490-1510); 2:5’-ACCAATTTAT-GCCTACAGCCTC-3’ (nt1798-1777). The procedures followed instructions from the manufacturers.

### 3.9. Liver Function Test

Serum ALT, total bilirubin (TBil) and albumin (ALB) were determined with Hitachi 7600 automatic biochemical analyzer.

### 3.10. HBV DNA

HBV DNA was assayed using real time fluorescence quantitative PCR, the reagents were purchased from Kehua, Shanghai, China.

### 3.11. Statistical Analysis

SPSS 12.0 was used for statistical analysis. Chi-square was applied for categorical data and t-test was used for comparisons of numerical data which are expressed as mean ± standard deviation (SD) between the two groups. The differences were considered statistically significant when P < 0.05.

## 4. Results

### 4.1. Characteristics of HBV Genotype Distribution of CHB Patients

Of the 150 CHB patients, 70 cases were infected with HBV genotype C (46.67%), 79 cases were infected with HBV genotype B (52.67%), one case (0.67%) was infected with both genotype B and C (the mixed type).

### 4.2. Relation between HBV Genotypes B, C, Gender and Age

Of the 70 cases infected with HBV genotype C, 50 (71.43%) were male and 20 (28.57%) were female; of the 79 cases infected with HBV genotype B, 56 (70.89%) were male, 23 (29.11%) were female, χ^2^ = 0.01, P > 0.05. Average age of patients infected with genotype C was (35.39 ± 8.34) years, average age of patients infected with HBV genotype B was (34.82 ± 8.13) years, t = 0.42, P > 0.05. The patient infected with the mixed genotype B and C was a 47-year-old female.

### 4.3. Relation between HBV Genotypes B, C and Tfh

**Figure 1:**
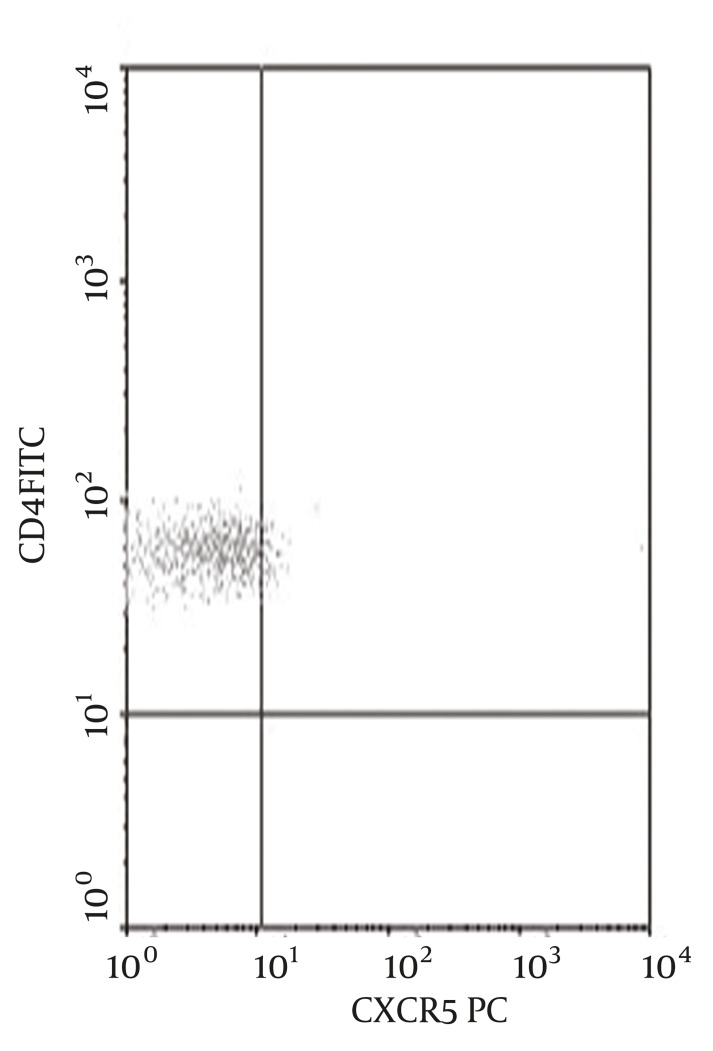
Flow Cytometric Diagram of Peripheral Blood Tfh

Peripheral blood Tfh level of 150 CHB patients (percentage in CD4 + T lymphocytes was 4.95 ± 2.35%), which was higher than that of the 30 normal controls (percentage in CD4+ T lymphocytes was 2.67 ± 0.97%), t = 6.15 , P < 0.01, peripheral blood Tfh level of patients infected with HBV genotype C (percentage in CD4+ T lymphocytes was 3.85 ± 2.43%) lower than that of patients infected with HBV genotype B (percentage in CD4 + T lymphocytes was 5.91 ± 1.84%), t = 4.76 P < 0.01. For flow cytometric diagram of peripheral blood Tfh see Figure 1.

### 4.4. Relation between HBV Genotypes B, C and IL-21

Peripheral blood IL-21 level of the 150 CHB patients (30.44 ± 19.94 ng/L) was significantly lower than that of the 30 normal controls (75.93 ± 25.88 ng/L), t = 9.98, P < 0.01, peripheral blood IL-21 level (15.80 ± 2.44ng/L) of patients infected with HBV genotype C was significantly lower than that of patients infected with HBV genotype B (43.26 ± 19.70ng/L), t = 7.96, P < 0.01.

### 4.5. Relation between HBV Genotypes B, C and HBV Specific CTL 

**Figure 2:**
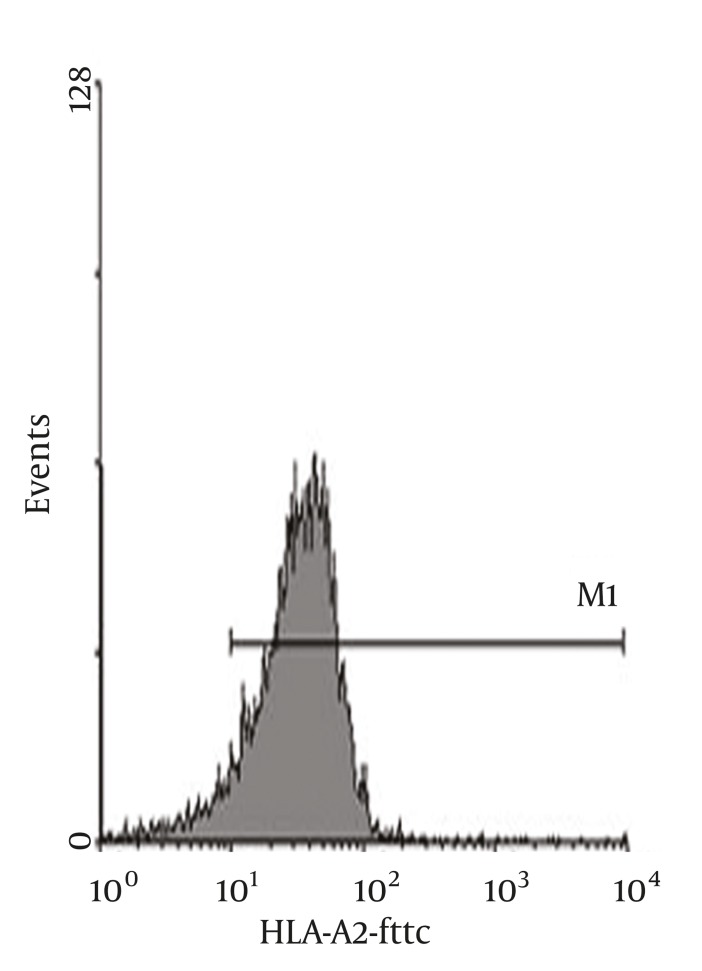
Flow Cytometric Diagram of HLA-A2 Allelotype

**Figure 3:**
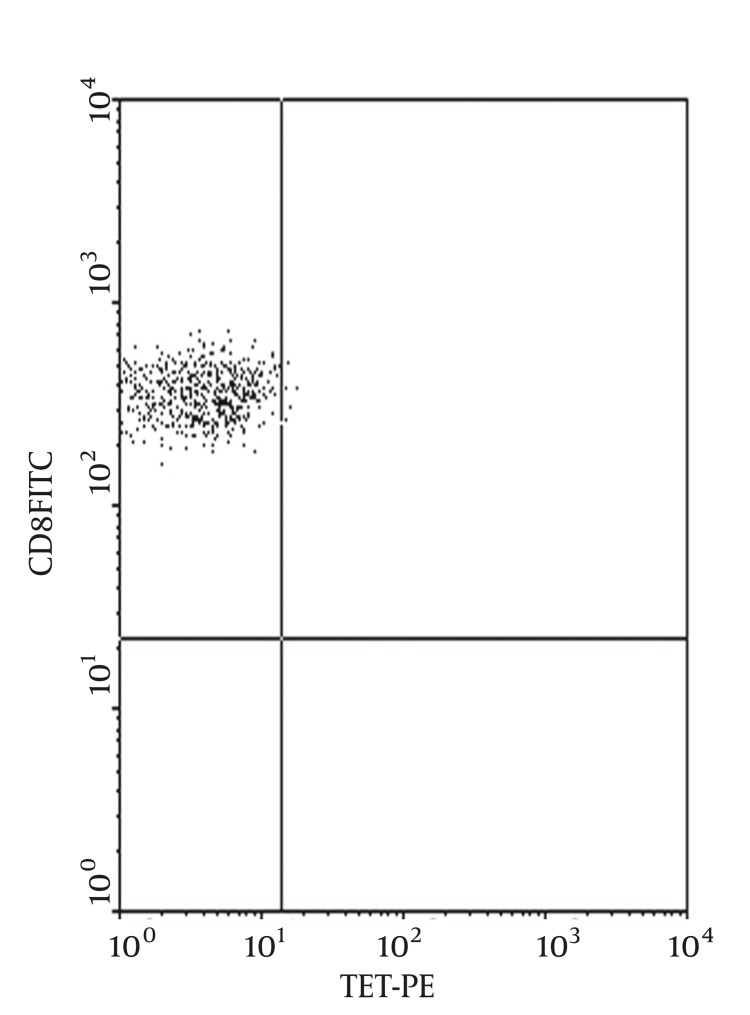
Flow Cytometric Diagram of HBV Specific CTL

Peripheral blood HBV specific CTL level of the 150 CHB patients was 0.30 ± 0.08%, peripheral blood HBV specific CTL of patients infected with HBV genotype C (0.23 ± 0.03%) was significantly lower than that of patients infected with HBV genotype B (0.37 ± 0.03%), t = 19.66, P < 0.01(For flow cytometric diagram of HLA-A2 allelotype see Figure 2, and for flow cytometric diagram of HBV specific CTL see Figure 3).

### 4.6. Relation between HBV Genotypes B, C and Nonspecific CTL

Peripheral blood nonspecific CTL level of the 150 CHB patients was 18.01 ± 2.34%, which was significantly higher than that of the 30 normal controls (15.83 ± 4.99%), t=2.98, P < 0.01, while peripheral blood nonspecific CTL of patients infected with HBV genotype C (19.72 ± 1.07%) was significantly higher than that of the patients infected with HBV genotype B (16.65±2.21%), t = 6.88, P < 0.01. The values of Tfh, IL-21, HBV specific CTL, and HBV-nonspecific CTL in HBV genotype C and B infected patients are listed in Table 1.

**Table 1:** Comparison of Peripheral Blood Tfh, IL-21, HBV Specific CTL and Nonspecific CTL between Patients Infected With HBV Genotypes B and C

Group	No.	Tfh	IL-21, ng/L	HBV- Specific CTL	HBV-Nonspecific CTL
**Genotype C, Mean±SD **	70	3.85 ± 2.43	15.80 ± 2.44	0.23 ± 0.03	19.72 ± 1.07
**Genotype B, Mean±SD**	79	5.91 ± 1.84	43.26 ± 19.70	0.37 ± 0.03	16.65 ± 2.21
**t value**		4.76	7.96	19.66	6.88
**P value**		＜ 0.01	＜ 0.01	＜ 0.01	＜ 0.01

Abbreviation: CTL, cytotoxic T lymphocytes

### 4.7. Relation between HBV Genotypes B, C and HBV DNA Level

HBV DNA level of the 150 CHB patients was 5.91 ± 1.12 log10 copies/ml, serum HBV DNA level of patients infected with HBV genotype C was (6.87 ± 0.35 log10 copies/ml), significantly higher than that of patients infected with HBV genotype B (5.03 ± 0.55 log10 copies/ml), t = 10.87, P < 0.01).

### 4.8. Relation between HBV Genotypes B, C and ALT and TBil

Mean ALT of the 150 CHB patients was 377.22 ± 133.01U/L, ALT of patients infected with HBV genotype C was 500.35 ± 81.81 U/L, higher than that of patients infected with genotype B (269.,51 ± 46.62 U/L), t = 13.86 , P < 0.01. TBil of the 150 CHB patients was 35.20 ± 11.51 μmol/L. TBil of patients infected with genotype C was 43.44 ± 12.13 μmol/L, higher than that of patients infected with genotype B (28.00 ± 3.05 μmol/L), t = 6.98 , P < 0.01. Relation between HBV B, C genotypes and levels of serum HBV DNA, ALT and TBil has been illustrated in Table 2.

**Table 2:** Comparison of Serum HBV DNA, ALT and TBil Levels between CHB Patients Infected With Genotype B and C

Group	No.	HBV DNA, log10 copies/ml	ALT, U/L	TBil, μmol/L
**Genotype C, Mean±SD**	70	6.87 ± 0.35	500.35 ± 81.81	43.44 ± 12.13
**Genotype B, Mean±SD**	79	5.03 ± 0.55	269.51 ± 46.62	28.00 ± 3.05
**t value**		10.87	13.86	6.98
**P value**		＜ 0.01	＜ 0.01	＜ 0.01

Abbreviations: ALT, alanine transminase; CHB, chronic hepatitis B; TBil, total bilirubin

## 5. Discussion

The patients infected with genotype C had a higher level of HBV DNA, suffer more serious damage to liver function, ALT fluctuating repeatedly or elevated persistently, and a lower response rate to antiviral therapy as compared with CHB patients infected with HBV genotype B. Persistent response of patients infected with HBV genotype C to the antiviral therapy was significantly lower than that of patients infected with HBV genotype B. HBV genotype C is significantly more prevalent than HBV genotype B in patients with cirrhosis or cancer of the liver (16, 17), suggesting that active and effective antiviral therapy for patients infected with HBV genotype C may be able to reduce occurrence of cirrhosis or cancer of the liver. However, at present, how to improve the effect of antiviral therapy and prognosis of CHB patients infected with genotype C of HBV is an urgent but difficult-to-solve problem, while the effect of antiviral therapy and sustained response are closely related to immune functions of the patient. Tfh is a T cell subgroup mainly responsible for helping the B cell (9). There are only a few reports on changes and functions of Tfh in patients with hepatitis B virus infection. Since Tfh can produce and secrete IL-21 (10, 11), which can increase proliferation of virus specific CTL (12, 14), there may be a certain relationship between Tfh and infection with hepatitis B virus. The results of the present study confirmed this hypothesis. It was found that peripheral blood Tfh level of CHB patients was higher than that of the healthy persons and there was a significant difference between genotype B and C of HBV in thiscontext. Different HBV genotypes have different biological characteristics, which can influence expression of viral antigens and immune functions, therefore, different genotypes of the virus may result in different clinical manifestations (18). The difference in sequence of S protein amino acid of HBV genotype C is more remarkable than that of HBV genotype B, and T cell locus of different small S protein amino acid sequences of HBV genotype C has changed significantly. It is possible that HBV genotype C is prone to variation as compared with HBV genotype B, resulting in higher rate of T cell locus change of S protein amino acid sequence of HBV genotype C than that of genotype B. Such changes may lead to weakened cell-mediated immune response of the body and affect HBV elimination (19). The present study showed that HBV genotypes B and C infection may lead to development of different biological characteristics in the patients, the rate of change in T cell locus of S protein amino acid sequence of HBV genotype C may be higher than that of genotype B, which may result in altered identification and response of Tfh to HBV genotype C and B infection, induction of Tfh by HBV genotype C may be lower than that by genotype B, therefore Tfh level of patients infected with genotype C is lower than that of the patients infected with genotype B. Because Tfh can produce and secrete IL-21, IL-21 can increase proliferation of virus specific CTL. In an experimental animal model of acute HBV infection (20), liver tissues of adult rats showed higher IL-21 expression related to hepatitis B virus compared with young rats. In mankind, in blood samples from adults with acute HBV infection who had virus elimination, IL- 21 level increased significantly, suggesting that IL-21 may be a part of the immune response to HBV. The results of the present study showed that lower level of peripheral blood IL-21 of CHB patients than normal controls may also suggest that decrease of IL- 21 level may be one of the causes that result in chronic infection with HBV. As Tfh level of patients infected with HBV genotype C was lower than that of patients infected with HBV genotype B, which may have resulted in lower level of IL-21 of patients with genotype C than that of genotype B. IL-21 can increase proliferation of virus specific CTL, resulting in lower proliferation and lower level of HBV specific CTL in patients infected with HBV genotype C than that of patients infected with HBV genotype B, specific cellular immune response of the body to the virus may be an important factor to eliminate the virus from the body (3), because lower level of HBV specific CTL of patients infected with HBV genotype C may have resulted in their higher level of HBV DNA than that of patients infected with HBV genotype B, more serious damage to liver function than in patients infected with HBV genotype B. When HBV specific CTL response is inadequate, HBV cannot be cleared effectively, and nonspecific CTL may start and result in inflammation of the liver and damage to the liver cells (8), while HBV specific CTL of patients infected with HBV genotype C is lower, when nonspecific CTL response starts, its level is higher than that of patients infected with HBV genotype B, resulting in higher level of nonspecific CTL and poorer liver functions as compared with those of patients infected with HBV genotype B. The limitations of the present study include relatively smaller sample size, less strictly matched subjects between the genotypes B and C infected groups, no determination and detailed study of Tfh, IL-21 and HBV specific CTL. Both the results and limitations of the present study warrant larger-scale, more strictly designed clinical and laboratory studies to further clarify the relationship between the genotypes and Tfh cell responses and severity of CHB and roles of these factors in treatment and prognosis of CHB. In conclusion, the present study showed that peripheral blood HBV specific CTL of CHB patients infected with HBV genotype C was significantly lower than that of genotype B, and its mechanism may be associated with lower level of Tfh than that of patients infected with HBV genotype B. Compared with CHB patients infected with genotype B of HBV, higher levels of serum HBV DNA, ALT and TBil of patients infected with HBV genotype C may be related to their lower level of peripheral blood Tfh, which may result in lower IL-21, and it may result in lower HBV specific CTL.
